# A novel immune prognostic index for stratification of high-risk patients with early breast cancer

**DOI:** 10.1038/s41598-020-80274-5

**Published:** 2021-01-08

**Authors:** Hannah Lee, Mi Jeong Kwon, Beom-Mo Koo, Hee Geon Park, Jinil Han, Young Kee Shin

**Affiliations:** 1 Interdisciplinary Program in Bioinformatics, College of Natural Sciences, Seoul National University, Seoul, 41566 Republic of Korea; 2 College of Pharmacy, Kyungpook National University, Daegu, 41566 Republic of Korea; 3 Research Institute of Pharmaceutical Sciences, Kyungpook National University, Daegu, 41566 Republic of Korea; 4 Department of Molecular Medicine and Biopharmaceutical Sciences, Graduate School of Convergence Science and Technology, Seoul National University, Seoul, 08826 Republic of Korea; 5 Gencurix, Inc., Seoul, 08394 Republic of Korea; 6 Laboratory of Molecular Pathology and Cancer Genomics, Department of Pharmacy, College of Pharmacy, Seoul National University, 1 Gwanak-ro, Gwanak-gu, Seoul, 08826 Republic of Korea

**Keywords:** Biomarkers, Oncology

## Abstract

The prognostic value of current multigene assays for breast cancer is limited to hormone receptor-positive, human epidermal growth factor receptor 2-negative early breast cancer. Despite the prognostic significance of immune response-related genes in breast cancer, immune gene signatures have not been incorporated into most multigene assays. Here, using public gene expression microarray datasets, we classified breast cancer patients into three risk groups according to clinical risk and proliferation risk. We then developed the immune prognostic index based on expression of five immune response-related genes (*TRAT1*, *IL2RB, CTLA4*, *IGHM* and *IL21R*) and lymph node status to predict the risk of recurrence in the clinical and proliferation high-risk (CPH) group. The 10-year probability of disease-free survival (DFS) or distant metastasis-free survival (DMFS) of patients classified as high risk according to the immune prognostic index was significantly lower than those of patients classified as intermediate or low risk. Multivariate analysis revealed that the index is an independent prognostic factor for DFS or DMFS. Moreover, the C-index revealed that it is superior to clinicopathological variables for predicting prognosis. Its prognostic significance was also validated in independent datasets. The immune prognostic index identified low-risk patients among patients classified as CPH, regardless of the molecular subtype of breast cancer, and may overcome the limitations of current multigene assays.

## Introduction

Several multigene assays, including Oncotype DX^1^, MammaPrint^2^, PAM50 Prosigna^3^, and EndoPredict^4^ were developed to predict the risk of recurrence or response to adjuvant chemotherapy in those with early breast cancer. These assays provide additional prognostic value and support traditional clinical factors. Oncotype DX^5–7^ and MammaPrint^8^ in particular were validated to predict responses to adjuvant chemotherapy. Accumulating evidence supports the prognostic value and clinical utility of these assays; therefore, international guidelines suggest that the results of these assays can help clinicians decide whether adjuvant chemotherapy will be of benefit to those with early breast cancer^9,10^. However, the prognostic or predictive value of these assays is limited to hormone receptor-positive, human epidermal growth factor receptor 2-negative (HR+/HER2−) early breast cancer^11^. Moreover, they have limited prognostic ability for late recurrence (> 5 years after diagnosis)^12^ and there is considerable discordance among assays with respect to risk stratification^13^. Accordingly, improvements to existing multigene assays, or development of novel assays, are needed to ensure more accurate prediction of the risk of distant recurrence or responses to treatment.

Most current multigene assays for breast cancer rely on expression of hormone receptor or proliferation-related genes. Desmedt et al. revealed that different breast cancer subtypes show different prognostic gene signatures, and that the strong prognostic impact of proliferation-related gene signatures is restricted to estrogen receptor-positive, HER2-negative (ER+/HER2−) breast cancer^14^. Proliferation-based gene signatures are less prognostic for late recurrence, but strongly prognostic for early recurrence, of ER+/HER2− breast cancer^15^. However, numerous studies show that immune gene signatures are crucial for the prognosis of HR- breast cancer^16–22^. The presence or high expression of immune-related genes is associated with favorable outcomes for patients with HR−/HER2+ or triple-negative breast cancer (TNBC, HR−/HER2−)^16–22^. Several molecular predictors of recurrence of HR− breast cancer based on prognostic immune gene signatures have been reported^16,17,20,23^. However, the prognostic or predictive significance of immune gene signatures with respect to HR+ breast cancer remains unclear. Of note, previous studies show that immune gene signatures can be prognostic for ER+ breast cancer. Schmidt et al. showed that the B cell metagene is independently associated with reduced risk of metastasis of lymph node-negative (LN−) breast cancer with high proliferative activity^24^. Similarly, we previously developed a novel prognostic model for LN− breast cancer based on the combination of proliferation-related genes and immunity-related genes^25^. Importantly, the study revealed that high proliferative activity is associated with an increased immune response in those with ER− or ER+ breast cancer, and that the positive prognostic value of immune response genes was true for LN− breast cancer, regardless of ER status. These results suggest that immune responses have a positive effect on the clinical outcome of fast-proliferating early breast cancer, regardless of ER status.

Despite the known prognostic or predictive significance of immune gene signatures in breast cancer, immune response-related genes have not been incorporated into commonly used commercial multigene assays. Recently, a multigene prognostic assay called Geneswell Breast Cancer Test (BCT), which includes the immune response-related *BTN3A2* (immunoglobulin superfamily related to T cell immune reaction), was developed and validated as prognostic for HR+/HER2− early breast cancer^26^. However, to date, there is no immune gene signature-based commercial assay that is prognostic for both HR+ and HR− breast cancer. Based on a previous study in which we showed a significant association between immune response-related genes and favorable outcomes in patients with highly proliferating tumors, regardless of ER status, we aimed to develop the immune gene-based prognostic model to further predict the risk of recurrence of high-risk early breast cancer, regardless of molecular subtype. Here, we first classified patients with four molecular subtypes into three risk groups according to clinical risk and proliferation risk. We then developed a novel prognostic model based on combined expression of five immune response-related genes and LN status and used it to predict the risk of recurrence in high-risk patients with early breast cancer.

## Results

### Classification of breast cancer patients into risk groups according to clinical risk and proliferation risk

The overall scheme of this study is presented in Supplementary Fig. S1. Among the 1327 patients in the five Gene Expression Omnibus (GEO) datasets, 916 with early breast cancer who were not treated with chemotherapy were included in the discovery dataset. Overall, 65.9% of patients (n = 604) were aged > 50 years (median, 57.5 years; range, 24–90), and most tumors were LN− (80.2%) and histologic grade 1 or 2 (70.9%) (Table 1). Most were of the HR+/HER2 subtype (64.7%, n = 593), followed by TNBC (20.6%, n = 189), HR+/HER2+ (10.1%, n = 93), and HR−/HER2+ subtype (4.5%, n = 41). The median follow-up period was 7.3 years.

**Table 1:** Clinical characteristics of the patients included in the discovery dataset.

	Total	HR+/HER2−	HR+/HER2+	HR−/HER2+	TNBC
	(n = 916)	(n = 593)	(n = 93)	(n = 41)	(n = 189)
	No. of patients (%)	No. of patients (%)	No. of patients (%)	No. of patients (%)	No. of patients (%)
**Age (years)**					
≤ 50	312 (34.1)	164 (27.7)	41 (44.1)	15 (36.6)	92 (48.7)
> 50	604 (65.9)	429 (72.3)	52 (55.9)	26 (63.4)	97 (51.3)
**Tumor size (cm)**					
≤ 2	507 (55.3)	312 (52.6)	46 (49.5)	12 (29.3)	137 (72.5)
2–5	396 (43.2)	272 (45.9)	44 (47.3)	29 (70.7)	51 (27.0)
> 5	13 (1.4)	9 (1.5)	3 (3.2)	0 (0.0)	1 (0.5)
**LN status**					
Negative	735 (80.2)	457 (77.1)	74 (79.6)	30 (73.2)	174 (92.1)
Positive	181 (19.8)	136 (22.9)	19 (20.4)	11 (26.8)	15 (7.9)
**Histologic grade**					
1&2	650 (70.9)	506 (85.3)	67 (72.0)	13 (31.7)	64 (33.8)
3	266 (29.0)	87 (14.7)	26 (28.0)	28 (68.3)	125 (66.1)

HER2, human epidermal growth factor receptor 2; HR, hormone receptor; LN, lymph node; TNBC, triple-negative breast cancer.

**Figure 1:**
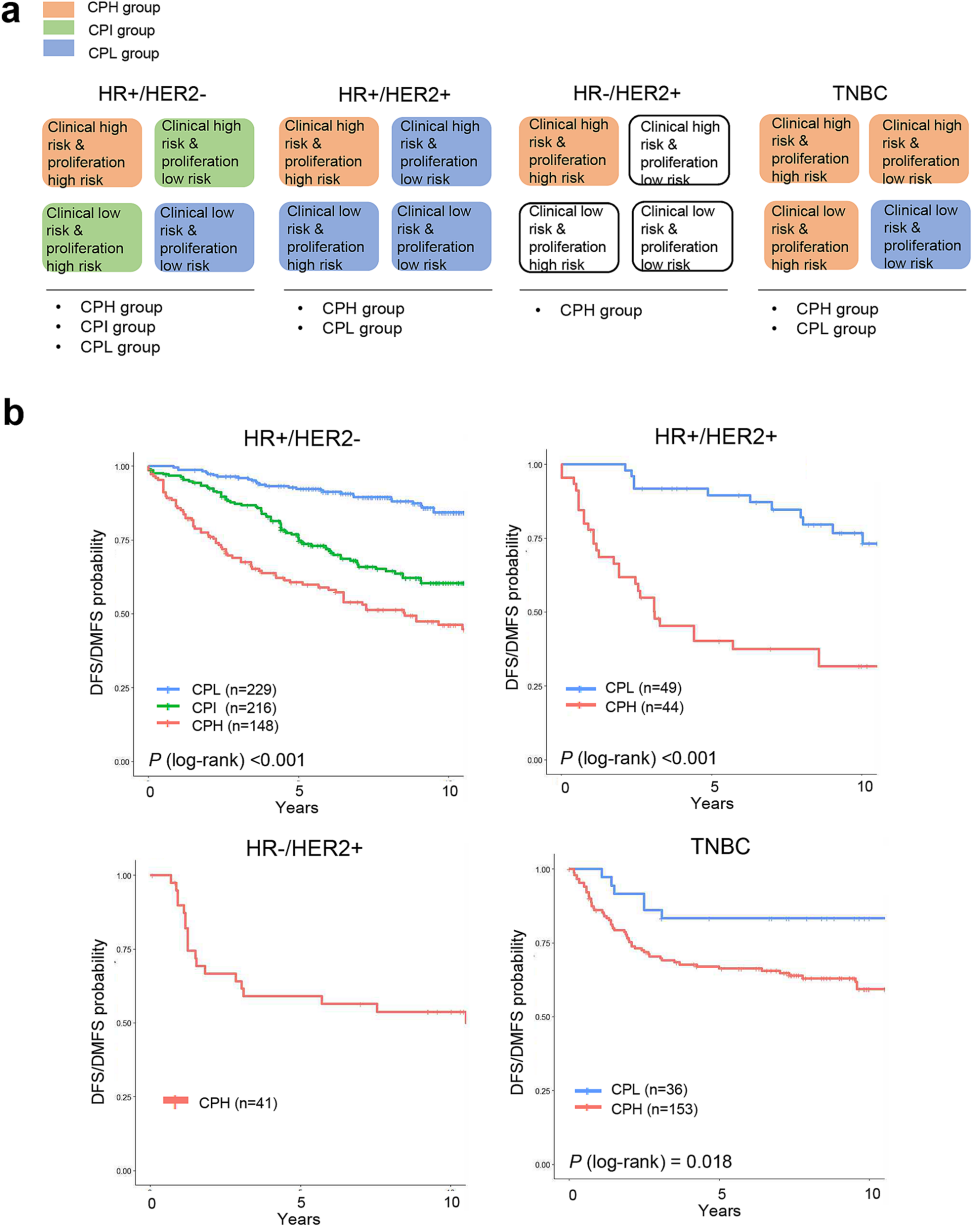
Stratification of patients in the discovery dataset according to clinical risk and proliferation risk. (**a**) Classification of patients with each molecular subtype of breast cancer according to clinical risk and proliferation risk. The four risk groups were regrouped into three risk groups: clinical and proliferation high-risk (CPH), intermediate-risk (CPI), and low-risk (CPL). (**b**) Kaplan–Meier plots for patients in the CPH, CPI, and CPL groups according to each molecular subtype of breast cancer. Differences in survival between groups were assessed using the log-rank test.

Patients with each subtype of breast cancer were first classified into four risk groups according to clinical risk and proliferation risk: (1) clinical high-risk and proliferation high-risk; (2) clinical high-risk and proliferation low-risk; (3) clinical low-risk and proliferation high-risk; and (4) clinical low-risk and proliferation low-risk groups. The four risk groups (assigned according to the clinical risk and proliferation risk) for each molecular subtype of breast cancer were further grouped into three risk groups based on Cox analysis outcomes: a clinical and proliferation high-risk (CPH) group; a clinical and proliferation intermediate-risk (CPI) group; and a clinical and proliferation low-risk (CPL) group. Patients with the HR+/HER2− subtype were assigned to the three risk groups, whereas patients in the HR+/HER2+ ubtype and TNBC subtype were assigned to only two risk groups (CPH and CPI); all HR−/HER2+ patients were assigned to the CPH group (Fig. 1a). The probability of disease-free survival (DFS) or distant metastasis-free survival (DMFS) of patients classified into the CPH group were significantly lower than those of patients classified into the CPI or CPL groups (Fig. 1b).

Moreover, the association between 110 immune response related-genes and the clinical outcomes of the CPH, CPI, and CPL groups were assessed with respect to each molecular subtype. In all CPH patients within each molecular subtype, expression of immune response-related genes was associated with favorable clinical outcomes. However, no significant immune response-related genes were associated with a favorable clinical outcome in the CPI and CPL groups. Based on these results, the CPH group was used to develop the novel immune gene-based prognostic model. 61.4% of patients in the CPH group were aged > 50 years and 286 (74.1%) patients had LN-tumors (Supplementary Table S1).

### Prognostic value of the immune prognostic index in the clinical and proliferation high-risk group

**Figure 2:**
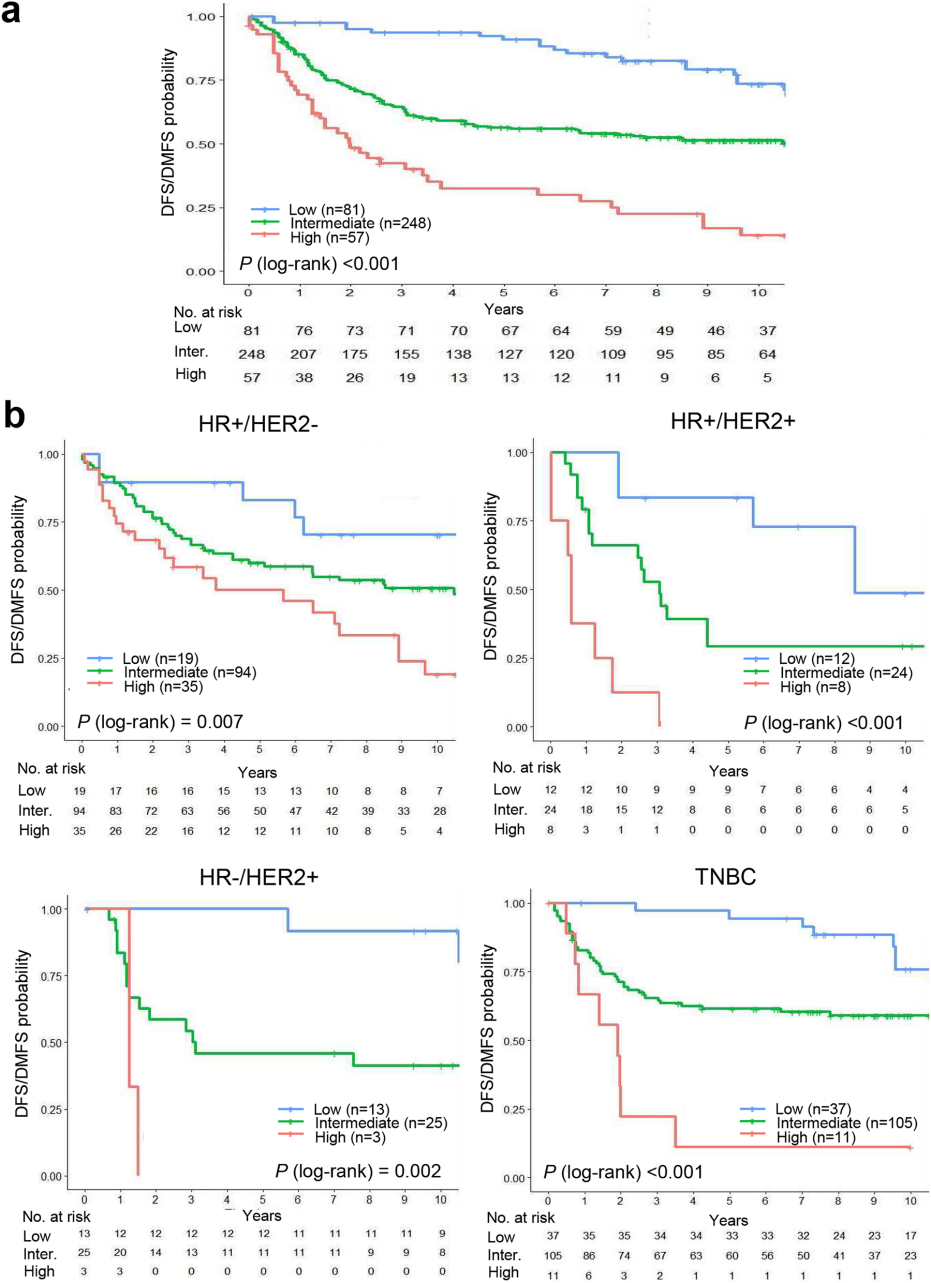
Kaplan–Meier plots of disease-free survival (DFS) or distant metastasis-free survival (DMFS) in the clinical and proliferation high-risk (CPH) group within the discovery dataset. (**a**) Total population and (**b**) subgroup analysis according to the molecular subtype (HR+/HER2−, HR+/HER2+, HR−/HER2+ breast cancer and TNBC). Patients were classified into three risk groups according to the immune prognostic index. Optimal cutoff points for the immune prognostic index used for risk classification were determined using maximally selected rank statistics.

The immune prognostic index was developed based on the expression of the top five immune response-related genes (Table 2) in combination with LN status to predict a recurrence in the CPH group. To assess the prognostic value of the immune prognostic index in the CPH group, the 386 patients were stratified into three risk groups (low, intermediate, and high) using the optimal cutoff points determined by maximally selected statistics. Kaplan–Meier curves revealed a significant difference in DFS or DMFS between the groups categorized according to the immune prognostic index. The survival rates of patients in the high-risk (hazard ratio, 5.77; 95% confidence interval [CI], 3.40–9.80) and intermediate-risk (hazard ratio, 2.42; 95% CI, 1.52–3.86) groups were significantly lower than those of patients in the low-risk group (*P* < 0.001) (Fig. 2a). The 10-year DFS or DMFS rates for patients in the low-, intermediate- and high-risk groups were 73.4%, 51.3%, and 14.1%, respectively. Moreover, the immune prognostic index was significant for DFS or DMFS of patients with any of the four molecular subtypes of breast cancer (*P* = 0.007 for HR+/HER2−; *P* < 0.001 for HR+ HER2+ and TNBC; *P* = 0.002 for HR−/HER2+) (Fig. 2b).

**Table 2:** The five prognostic immune response related genes used to calculate the immune prognostic index.

Gene group	Gene symbol	Full name	Gene ontology term
Immune response	*TRAT1*	T cell receptor associated transmembrane adaptor	Adaptive immune response; negative regulation of receptor recycling
	*IL21R*	Interleukin 21 receptor	Natural killer cell activation; interleukin-21-mediated signaling pathway
	*IGHM*	Immunoglobulin heavy constant mu	Adaptive immune response; phagocytosis
	*CTLA4*	Cytotoxic T lymphocyte-associated protein 4	Adaptive immune response; cellular response to DNA damage stimulus
	*IL2RB*	Interleukin 2 receptor subunit beta	MAPK cascade; protein complex assembly

Next, we analyzed the association between clinical variables, the immune prognostic index, and clinical outcomes. Univariate analysis revealed that the immune prognostic index (as a risk group or continuous variable) and LN status were significantly associated with risk of recurrence or distant metastasis (*P* < 0.001) (Table 3). Importantly, the immune prognostic index retained its statistical significance in multivariate analysis, indicating that it was an independent prognostic factor in the CPH group (Table 3).

**Table 3:** Univariate and multivariate analyses of the immune prognostic index and clinicopathological variables in the discovery dataset.

	Univariate analysis				Multivariate analysis				Multivariate analysis		
	Hazard ratio	95% CI	*P* value		Hazard ratio	95% CI	*P* value		Hazard ratio	95% CI	*P* value
No. of patients (n = 386)				No. of patients (n = 386)				No. of patients (n = 386)			
No. of event (n = 181)				No. of event (n = 181)				No. of event (n = 181)			
**Immune prognostic index**				**Immune prognostic index**				**Immune prognostic index**			
Continuous				Continuous				Risk groups			
As score increases	1.46	1.30–1.65	**< 0.001**	As score increases	1.40	1.21–1.61	**< 0.001**	Low	1.00		
Risk groups								Intermediate	2.33	1.46–3.73	**< 0.001**
Low	1.00							High	4.81	2.68–8.63	**< 0.001**
Intermediate	2.42	1.52–3.86	** < 0.001**								
High	5.77	3.40–9.80	**< 0.001**								
**Clinical variables**				**Clinical variable**				**Clinical variable**			
Age (years)				LN status				LN status			
≤ 50	1.00			Negative	1.00			Negative	1.00		
> 50	0.9	0.67–1.21	0.495	Positive	1.25	0.86–1.81	0.234	Positive	1.31	0.91–1.88	0.140
Tumor size (cm)											
≤ 2	1.00										
> 2	0.75	0.55–1.02	0.068								
LN status											
Negative	1.00										
Positive	2.02	1.48–2.76	**< 0.001**								
Histologic grade											
1&2	1.00										
3	0.80	0.60–1.08	0.146								

CI, confidence interval; LN, lymph node.

*P* values < 0.05 are marked in bold.

**Figure 3:**
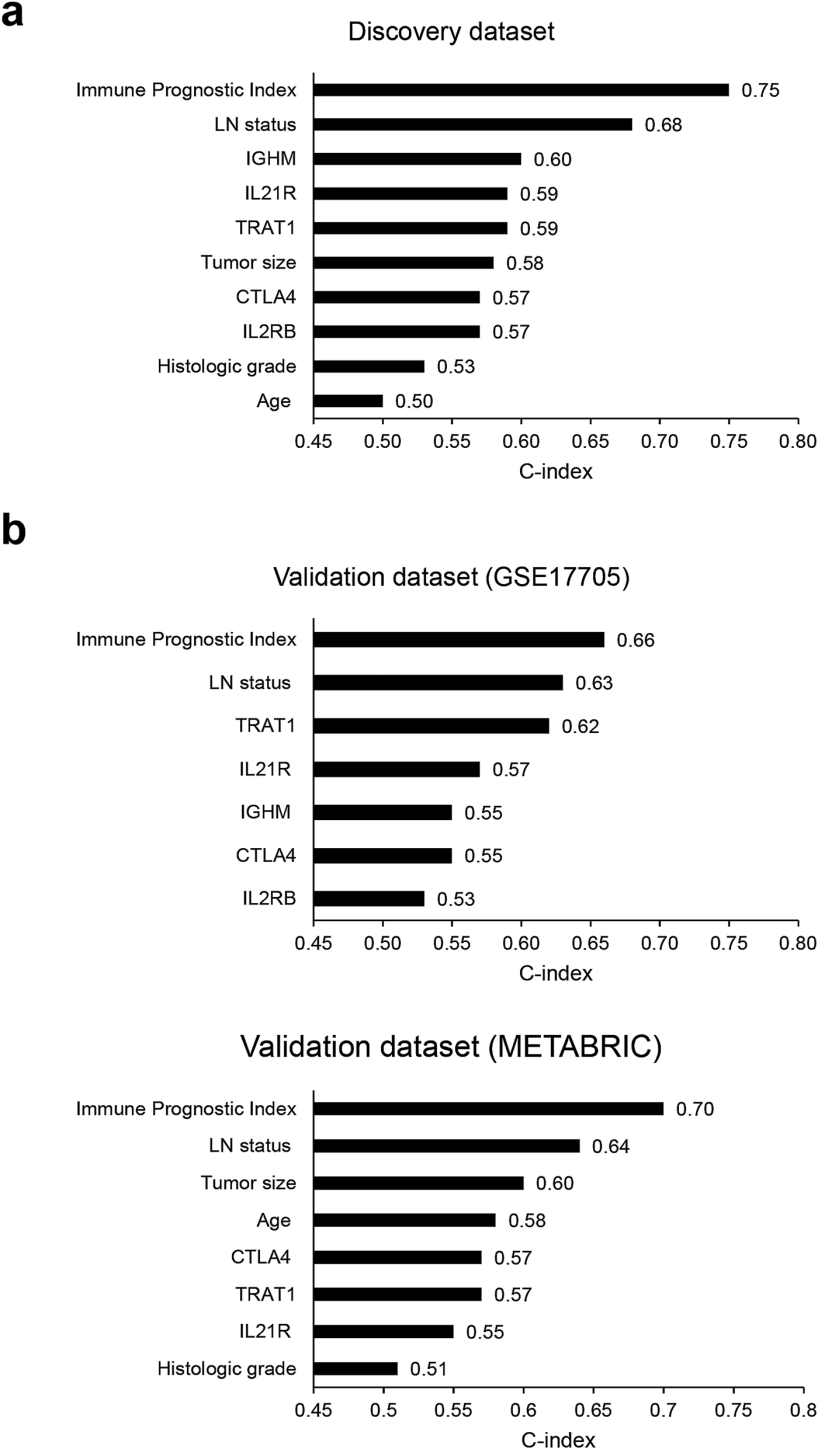
The C-index comparing the prognostic performance of the immune prognostic index for predicting patient survival with that of clinicopathological variables. (**a**) Discovery dataset (**b**) Validation datasets (top, GSE17705; bottom, METABRIC). Values on the x-axis indicate C-index estimates for clinical/gene variables and the immune prognostic index.

The C-index was used to compare the prognostic performance of the immune prognostic index compared with that of clinical/gene variables used to develop prognostic model. As shown in Fig. 3a, the immune prognostic index had the highest C-index (0.75). These results illustrate that the immune prognostic index (based on the combination of expression of five prognostic immune genes and LN status) is superior to other clinical/gene variables used to predict recurrence or distant metastasis in those with high-risk early breast cancer.

### Validation of the immune prognostic index using independent datasets

**Figure 4:**
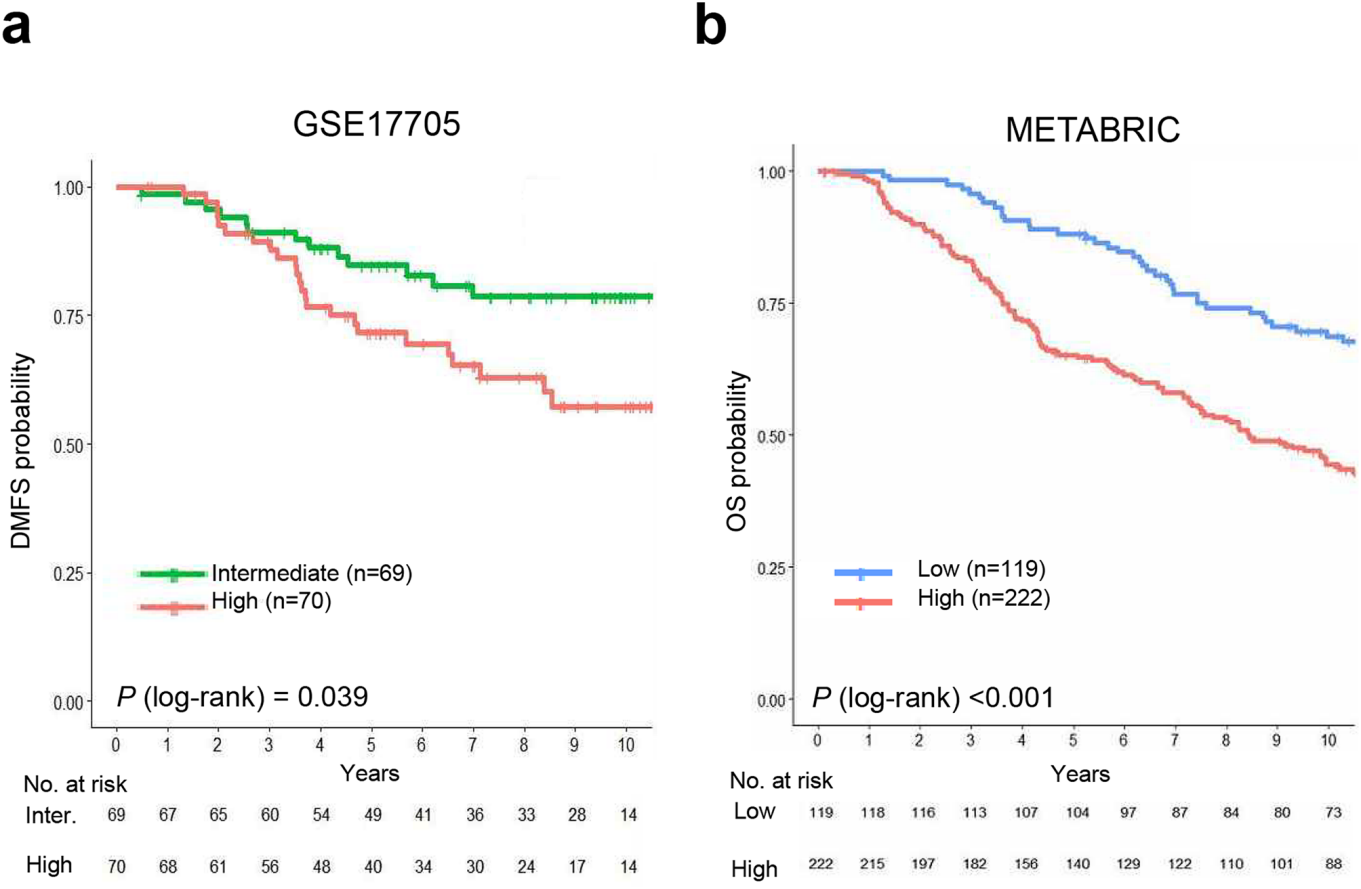
Kaplan–Meier plot of distant metastasis-free survival (DMFS) or overall survival (OS) for the validation datasets. (**a**) GSE17705 dataset and (**b**) METABRIC dataset. Patients in GSE17705 were stratified into intermediate- and high-risk groups, and patients in the METABRIC dataset were stratified into low- and high-risk groups, according to the immune prognostic index. Optimal cutoff points for the immune prognostic index used for risk classification were determined using maximally selected rank statistics.

Next, we validated the prognostic significance of the immune prognostic index using independent datasets (GSE17705 and Molecular Taxonomy of Breast Cancer International Consortium [METABRIC] datasets). Similar to discovery dataset, a higher percentage of patients with LN− tumors than those with LN+ tumors was included in these validation datasets (Supplementary Table S1). Patients in the CPH group were stratified into two risk groups according to the optimal cutoff of the immune prognostic index. In the GSE17705 dataset, there was a significant difference in the DMFS of the intermediate- and high-risk groups. Patients classified as high risk had a significantly lower probability of DMFS than patients in the intermediate-risk group (*P* = 0.039) (Fig. 4a). Furthermore, multivariate analysis revealed that the immune prognostic index (as a continuous variable) is an independent prognostic factor (hazard ratio, 1.40; 95% CI, 1.21–1.61; *P* < 0.001) (Table 4). The C-index was highest for the immune prognostic index (0.66), indicating that the index is better than clinical variables for predicting prognosis (Fig. 3b).

**Table 4:** Univariate and multivariate analyses of the immune prognostic index and clinicopathological variables in the validation datasets (GSE17705 and METABRIC).

Univariate analysis				Multivariate analysis				Multivariate analysis			
	Hazard ratio	95% CI	*P* value		Hazard ratio	95% CI	*P* value		Hazard ratio	95% CI	*P* value
No. of patients (n = 139)				No. of patients (n = 139)				No. of patients (n = 139)			
No. of events (n = 40)				No. of events (n = 40)				No. of events (n = 40)			
**Immune prognostic index**				**Immune prognostic index**				**Immune prognostic index**			
Continuous				Continuous				Risk groups			
As score increases	1.59	1.03–2.44	**0.035**	As score increases	1.40	1.21–1.61	**< 0.001**	Intermediate	1.00		
Risk groups								High	1.83	0.58–5.73	0.303
Intermediate	1.00										
High	1.94	1.02–3.69	**0.043**								
**Clinical variables**				**Clinical variable**				**Clinical variable**			
LN status				LN status				LN status			
Negative	1.00			Negative	1.00			Negative	1.00		
Positive	1.78	0.95–3.33	**0.072**	Positive	1.25	0.86–1.81	0.234	Positive	1.08	0.35–3.30	0.897
No. of patients (n = 341)				No. of patients (n = 341)				No. of patients (n = 341)			
No. of events (n = 226)				No. of events (n = 226)				No. of events (n = 226)			
**Immune prognostic index**				**Immune prognostic index**				**Immune prognostic index**			
Continuous				Continuous				Risk groups			
As score increases	1.32	1.19–1.48	**< 0.001**	As score increases	1.45	1.13–1.87	**0.004**	Low	1		
Risk groups								High	1.85	1.30–2.64	**< 0.001**
Low	1										
High	2.16	1.60–2.91	**< 0.001**								
**Clinical variables**				**Clinical variables**				**Clinical variables**			
Age (years)				Tumor size (cm)				Tumor size (cm)			
≤ 50	1			≤ 2	1			≤ 2	1		
> 50	1.12	0.72–1.74	0.618	> 2	1.58	1.12–2.23	**0.001**	> 2	1.57	1.11–2.22	**0.010**
Tumor size (cm)				LN status				LN status			
≤ 2	1			Negative	1			Negative	1		
> 2	1.66	1.17–2.33	**0.004**	Positive	0.76	0.42–1.41	0.390	Positive	1.24	0.90–1.69	0.185
LN status											
Negative	1										
Positive	1.74	1.33–2.26	**< 0.001**								
Histologic grade											
1&2	1										
3	0.86	0.64–1.14	0.295								

CI, confidence interval; GEO, Gene Expression Omnibus; LN, lymph node; METABRIC, Molecular Taxonomy of Breast Cancer International Consortium.

*P* values < 0.05 are marked in bold.

Similar results were observed for the METABRIC dataset. The 341 patients with stage I and II breast cancer were classified into low- or high-risk groups according to the immune prognostic index. However, because two immune response-related genes (*IGHM* and *IL2RB*) were not included in the METABRIC dataset, only three genes (*TRAT1*, *IL21R*, and *CTLA4*) were used to calculate the immune prognostic index. Survival analysis revealed that the immune prognostic index was prognostic for overall survival (OS) (*P* < 0.001) (Fig. 4b). It retained prognostic significance in multivariate analysis (*P* = 0.004 as a continuous variable; *P* < 0.001 as a risk group) (Table 4). Subgroup analysis of the METABRIC dataset according to molecular subtype showed that the immune prognostic index was prognostic for HR+/HER2+, HR−/HER2+, and TNBC (*P* < 0.05), but only not prognostic for HR+/HER2− (*P* = 0.24) breast cancer (Supplementary Fig. S2).

Although the immune prognostic index was developed to predict the risk of recurrence in patients with early breast cancer who were not treated with adjuvant chemotherapy, we also tested its ability to predict the prognosis in the datasets including chemotherapy-treated patients (in datasets GSE3494, GSE21653 and GSE42568). We found a significant difference in OS (*P* = 0.027 for GSE3494) or DFS (*P* = 0.007 for GSE21653 and 42568) between the two risk groups (Supplementary Fig. S3a). Indeed, the index (as continuous variable) was an independent prognostic factor (Supplementary Table S2). The C-index of the immune prognostic index (0.80) was higher than that of other clinical/gene variables (Supplementary Fig. S3b).

### Comparison of the immune prognostic index with other immune gene signatures

We compared the prognostic value of the immune prognostic index with other immune gene signatures for breast cancer including HRneg/Tneg signature linked to immune/inflammatory cytokine regulation (*ABO*, *CLIC5*, *CXCL13*, *EXOC7*, *HAPLN1*, *MATN1*, *PRRG3*, *PRTN3*, *RFX7*, *RGS4*, *RPS28*, *SSX3*, *ZNF3*, *HRBL*)^16^ and B cell response genes (*IGK@[IGKC]*, *GBP1*, *STAT1*, *IGLL5*, and *OCLN*)^27^ in the discovery and METABRIC dataset. Univariate and multivariate analysis showed that the immune prognostic index was an independent prognostic factor, while two immune gene signatures were not significant (Supplementary Table S3). The immune prognostic index also had a higher C-index than those of other signatures in discovery and METABRIC dataset, respectively (Supplementary Fig. S4).

## Discussion

Here, we developed and validated a novel immune gene-based prognostic index to predict recurrence or distant metastasis in high-risk patients with early breast cancer. First, we identified the most significant immune response-related genes associated with clinical outcome in a pre-specified CPH subgroup and then developed a novel immune prognostic index for this subgroup based on a combination of five immune response-related genes and LN status. When patients in the CPH group were classified into three risk groups according to the immune prognostic index, we found that the probability of DFS or DMFS of patients classified as intermediate or low risk were significantly higher than those of patients in the high-risk group. Multivariate analysis identified the immune prognostic index (as a continuous or categorical variable) as an independent prognostic factor. The prognostic value of the immune prognostic index was also validated in independent datasets. These results demonstrate that the immune gene-based model is prognostic for recurrence in the high-risk subgroup of all subtypes of breast cancer including HR+ and HR− breast cancer.

Given that the prognostic significance of immune gene signatures was demonstrated mainly for HR- breast cancer, it is important to ascertain whether immune genes may be prognostic for highly proliferating HR+ breast cancers. The immune prognostic index identified low-risk or intermediate-risk patients within the high-risk group; these patients may not benefit from adjuvant chemotherapy. These results are in line with those of our previous study showing the positive association of immune response-related genes with clinical outcomes in highly proliferating tumors, regardless of ER status^25^. Moreover, our findings are supported by a recent study, showing that 17 immune gene signatures are prognostic for DMFS only in patients with ER- and highly proliferating breast cancers^20^. Importantly, comparative analysis of prognostic performance of our model and other immune gene signatures suggests that our immune prognostic index may be superior to other immune gene signatures including B cell response genes and genes related to immune/inflammatory cytokine regulation in predicting the prognosis of early breast cancer.

The five genes (*TRAT1*, *IL21R*, *IGHM*, *CTLA4*, and *IL2RB*) used in the immune prognostic index play roles in immune responses by regulating the function of T cells, B cells, natural killer (NK) cells, or interleukin signaling pathways. Here, we found that high expression of these immune response-related genes was associated with a favorable prognosis in the high-risk subgroup of patients with early breast cancer. These results are consistent with previous studies showing that *IGHM* (immunoglobulin heavy constant mu) gene expression correlates with a better prognosis for TNBC^28^, that immunostimulatory cytokine IL2 (interleukin 2) signaling through interaction with its receptor IL2RB (interleukin 2 receptor subunit beta) enhances the anti-tumor effects of NK cells^29^, and *TRAT1* (T cell receptor associated transmembrane adaptor 1) is positivity associated with survival of melanoma patients^30^. By contrast, IL21 (interleukin 21) and IL21R (interleukin 21 receptor) play a role in promoting migration and invasion of breast cancer^31^. Another study shows that higher expression of IL21R in patients with primary HER2+ breast cancer is associated with positive effects of trastuzumab with respect to DFS, suggesting a possible role of IL21R as predictive marker for anti-HER2^32^. The study also showed that IL21R expression by CD8+ T cells is required for antitumor immune response of anti-ErbB2 antibodies against HER2+ tumors; also, IL21 signaling via IL21R may increase trastuzumab efficacy. Importantly, CTLA-4 (cytotoxic T lymphocyte antigen 4) suppresses activation of cytotoxic T cells, thereby contributing to the evasion of anti-tumor immune responses^33^. Higher expression of CTLA-4 mRNA levels is associated with advanced stage and axillary LN metastasis in those with breast cancer^34^. Given the known role of CTLA-4, we were surprised to find that its expression was associated with a favorable prognosis. This may be due to CTLA-4 expression by lymphocytes. Yu et al. showed that CTLA-4 expression by lymphocytes is associated with a favorable prognosis, whereas its expression by tumor cells is related to a poor prognosis^35^. However, as the prognostic significance and role of these five genes in breast cancer are largely unknown, it is notable that this study suggests that their expression is associated with a favorable prognosis in the high-risk subgroup with early breast cancer.

Current multigene assays are based on expression of proliferation-related genes, and their prognostic significance is limited to HR+/HER2− breast cancer. Proliferation-based gene signatures are strongly prognostic for ER+/HER2− breast cancer, but less so for other subtypes of breast cancer^15^. Our immune gene-based prognostic index may overcome the limitations of these current multigene assays and can be used alongside them to improve prognosis. However, there are some limitations. Owing to the lack of complete clinical information held in public microarray datasets, it was difficult to include a sufficient number of patients in the discovery and validation datasets. Also, there was a discrepancy between the discovery and validation datasets with respect to the optimal cutoff for classifying patients into risk groups. Moreover, only three genes (*TRAT1*, *IL21R*, and *CTLA4*) in the METABRIC dataset were used to calculate the immune prognostic index because two immune response-related genes (*IGHM* and *IL2RB*) were not included in this dataset. Finally, because the immune prognostic index was validated using public microarray datasets, its prognostic ability should be further validated by independent studies using samples derived from patient tissue.

We developed a novel prognostic model based on a combination of five immune response-related genes and LN status and used it to predict the risk of recurrence in a CPH group of patients with early breast cancer. The immune prognostic index identified low-risk patients among clinically high-risk patients with highly proliferating tumors belonging to all subtypes of breast cancer. Moreover, the immune prognostic index was an independent prognostic factor, with performance superior to that of clinical variables used to predict the risk of recurrence. Its prognostic significance was also validated using independent datasets. Thus, the immune prognostic index may be used to provide additional prognostic information and to support current multigene assays used to identify low-risk patients who do not require adjuvant chemotherapy for early breast cancer, regardless of subtype.

## Methods

### Public microarray data mining and analysis

Affymetrix microarray datasets of breast cancer, including clinical information such as molecular subtype, clinicopathological variables, treatments, and patient survival information were downloaded from the GEO (http://www.ncbi.nlm.nih.gov/geo). Five GEO datasets (GSE4922, GSE6532, GSE7390, GSE11121, and GSE31519) based on the Affymetrix HG-U133A (GPL96 platform) were pooled to generate a discovery dataset (Supplementary Table S4). Among the 1327 patients from five GEO datasets, those with early breast cancer who were not treated with adjuvant chemotherapy (treated with adjuvant hormone therapy alone or no adjuvant therapy) were included in the discovery dataset. Breast cancer was classified into four molecular subtypes according to ER or progesterone receptor (PR) and HER2 status: HR+/HER2− (ER+ or PR+ /HER2−), HR+/HER2+ (ER+ or PR+/HER2+), HR−/HER2+ (ER−/PR−/HER2+) breast cancer, and TNBC (ER−/PR−/HER2−). If datasets did not contain information about ER/PR and HER2 status, expression levels of the corresponding gene encoding each marker was used as described previously^36^. The downloaded datasets were log_2_ normalized. To reduce bias, genes with low variance were filtered out (interquartile range ≤ 0.5). Batch effects were removed using ComBat algorithm to reduce non-biological variations^37^.

In addition, independent GEO datasets based on Affymetrix HG-U133A (GPL96 platform) or HG-U133_Plus_2 (GPL570 platform), and Illumina bead-based METABRIC dataset were obtained; these were used as validation datasets (Supplementary Table S4). The METABRIC dataset was downloaded from the cBioPortal website (http://www.cbioportal.org).

### Identification of candidate prognostic proliferation and immune response-related genes in breast cancer

Candidate genes prognostic for breast cancer were identified using Cox regression analysis and gene pathway analysis of the discovery dataset. The top most significant genes with adjusted *P* values < 0.01 were selected by Cox regression analysis; these were annotated using DAVID bioinformatics resources^38,39^ and the gene annotation ‘topGO’ package in R (https://bioconductor.org/packages/release/bioc/html/topGO.html). In addition, a list of candidate prognostic proliferation-related genes^18,25^ and immune response-related genes^22,40^ was compiled from previous studies. Based on the data analysis and literature search, 37 proliferation-related genes and 110 immune response-related genes were selected as candidate prognostic genes (Supplementary Table S5). Most of immune response-related genes displayed a higher expression in patients with favorable prognosis compared with those with poor prognosis. Higher expression of immune response-related genes showed a tendency for being associated with favorable prognosis (Supplementary Table S6), whereas high expression of proliferation-related genes was related to poor clinical outcome.

### Stratification of patients according to clinical risk and proliferation risk

Clinical risk was assessed based on histologic grade, tumor size, and LN status using a modified version of Adjuvant! Online, as described previously^8^. Patients with higher histologic grade, larger tumor size, and positive LN status were classified as clinical high risk. To assess proliferation risk, prognostic proliferation-related genes were selected from the 37 candidate genes in the discovery dataset using multivariate analysis. Multivariate analysis of the discovery dataset identified ten proliferation-related genes (*BUB1B, RRM2, KIF18B, PTTG1, MELK, CDK1, FOXM1, TRIP13, RACGAP1,* and *KIFC1*) as independent prognostic indicators of DFS or DMFS (Supplementary Fig. S5). Based on expression of these ten proliferation-related genes, patients were classified into high- or low-risk groups. If expression of five or more genes was greater than the median expression level of all ten genes, the patient was assigned to the proliferation high-risk group; otherwise, the patient was assigned to the proliferation low-risk group.

### Development of a prognostic model based on expression of immune response-related genes and LN status

Lasso regression analysis was performed to identify the most significant immune response-related genes significantly associated with DFS or DMFS. The top five immune response-related genes (*TRAT1, IL21R, IGHM, CTLA4, and IL2RB*) with the lowest lasso regression coefficient (< − 0.05) (Table 2) were selected. Multivariate analysis of clinical variables (age, tumor size, histologic grade, and LN status) revealed that LN status was the most significant independent prognostic factor (data not shown). Therefore, expression of five immune response-related genes in combination with LN status was used to develop a prognostic score, referred to as the immune prognostic index, to predict a recurrence in the clinical and proliferation high-risk group. The coefficient values for each variable were calculated using Cox regression analysis, and the immune prognostic index was defined as a linear combination of these coefficients, which was used to predict the recurrence:

$$\begin{aligned} {\text{Immune}}\,{\text{Prognostic}}\,{\text{Index}}\, &= \,\left( { - 0.{3812}} \right)\,*\,\left( {TRAT1} \right)\, + \,\left( { - 0.{6586}} \right)\,*\,\left( {IL21R} \right)\, + \,\left( { - 0.{4732}} \right)\,*\,\left( {CTLA4} \right) \\ & \quad + \left( { - 0.{3642}} \right)\,*\,\left( {IGHM} \right)\, + \,\left( { - 0.{7}0{69}} \right)\,*\,\left( {IL2RB} \right)\, + \,\left( {0.{7}0{23}*{2}} \right)\,*\,\left( {\text{LN status}} \right) \\ \end{aligned}$$

where LN status is 0 (LN−) or 1 (LN+). A higher value for this index indicates a higher risk of recurrence or distant metastasis. The optimal cutoff for risk classification was determined using the maximally selected rank statistics in the ‘survminer’ R package^41^.

### Statistical analyses

DFS was defined as the time from the date of surgery to the date of relapse, including locoregional recurrence and distant metastasis. DMFS was defined as the time from the date of surgery for the primary tumor to the date of distant metastasis. OS was defined as the time from the date of surgery to the date of death. Univariate and multivariate analyses using Cox’s proportional hazard regression models were used to assess the association between clinical/gene variables and patient survival. All hazard ratios are reported with 95% CIs. The probability of DFS, DMFS, and OS was estimated using the Kaplan–Meier method and statistical differences in survival rates between groups were assessed using the log-rank test.

Lasso regression analysis to select the prognostic immune response-related genes was done using the ‘coxnet’ package in R (https://cran.r-project.org/web/packages/glmnet/vignettes/Coxnet.pdf). Lasso regression analysis performs regularization and feature selection by penalizing the coefficients of the input variables using optimal λ as a tuning parameter^42^. The prognostic performance of the immune prognostic index was compared with that of clinical/gene variables or other immune gene signatures using Harrell’s concordance index (C-index)^43^. R package ‘survcomp’ was used to calculate the C-index. *P* values < 0.05 were considered statistically significant. All statistical analyses were conducted using R 3.4.3 (http://r-project.org).

## Supplementary Information

Supplementary Information.

Supplementary Table.

## Data Availability

The datasets generated during and/or analysed during the current study are available in the GEO website (http://www.ncbi.nlm.nih.gov/geo) and cBioPortal website (http://www.cbioportal.org).
