# Supplementary material for: A novel immune prognostic index for stratification of high-risk patients with early breast cancer
Source: Sci Rep. 2021 Jan 8;11:128. doi: 10.1038/s41598-020-80274-5 (PMC7794340; doi:10.1038/s41598-020-80274-5)
Supplement: Supplementary file 1 — Supplementary Information. [file 41598_2020_80274_MOESM1_ESM.pdf]

Supplementary information

A Novel Immune Prognostic Index for Stratification of High-Risk Patients with Early Breast Cancer

Hannah Lee[+], Mi Jeong Kwon[+], Beom-Mo Koo, Hee Geon Park, Jinil Han, Young Kee Shin[*]

[+] Hannah Lee and Mi Jeong Kwon have contributed equally to this work.

[*]Corresponding author: Young Kee Shin (ykeeshin@snu.ac.kr)

Supplementary Tables

Supplementary Table S1. Patient characteristics of clinical and proliferation high-risk group in the discovery and validation datasets

	Discovery dataset	Validation datasets			
	No chemotherapy	No chemotherapy		Chemotherapy treated	
	Total (n=386) No. (%)	GSE17705 (n=139) No. (%)	METABRIC (n=341) No. (%)	GSE3494 (n=86) No. (%)	GSE21653&42568 (n=130) No. (%)
Age (years)					
≤50	149 (38.6)		36 (10.6)	23(26.7)	51(39.2)
>50	237 (61.4)		305 (89.4)	63(73.3)	79(60.8)
Tumor size (cm)					
≤2	152 (39.4)		120 (35.2)	19(22.1)	9(6.9)
2~5	228 (59.1)		217 (63.6)	64(74.4)	84(64.6)
>5	6 (1.6)		4 (1.1)	3(3.5)	37(28.5)
LN status					
Negative	286 (74.1)	76 (54.7)	207 (60.7)	38(44.2)	54(41.5)
Positive	100 (25.9)	63 (45.3)	134 (39.3)	48(55.8)	76(58.5)
Histologic grade					
1&2	152 (39.4)		92 (27.0)	41(47.7)	32(24.6)
3	234 (60.6)		249 (73.0)	45(52.3)	98(74.4)

Abbreviations : LN, lymph node status

Supplementary Table S2. Univariate and multivariate analyses of the immune prognostic index and clinicopathological variables in the validation datasets, including patients treated with adjuvant chemotherapy

GSE3494

	Univariate Analysis				Multivariate Analysis				Multivariate Analysis		
	Hazard Ratio	95% CI	*P* value		Hazard Ratio	95% CI	*P* value		Hazard Ratio	95% CI	*P* value
No. of patients (n=86)				No. of patients (n=86)				No. of patients (n=86)			
No. of events (n=33)				No. of events (n=33)				No. of events (n=33)			
**Immune prognostic index**				**Immune prognostic index**				**Immune prognostic index**			
Continuous				Continuous				Risk groups			
As score increases	2.24	1.41–3.57	**<0.001**	As score increases	2.73	1.13–6.58	**0.025**	Low	1		
Risk groups								High	1.61	0.46–5.55	0.454
Low	1										
High	2.81	1.08–7.28	**0.034**								
**Clinical variables**				**Clinical variable**				**Clinical variable**			
Age (years)				LN status				LN status			
≤50	1			Negative	1			Negative	1		
>50	0.76	0.37–1.56	0.448	Positive	0.68	0.16–2.88	0.604	Positive	2.1	0.80–5.56	0.134
Tumor size (cm)											
≤2	1										
>2	2.36	0.83–6.73	0.107								
LN status											
Negative	1										
Positive	2.74	1.30–5.80	**0.008**								
Histologic grade											
1&2	1										
3	0.97	0.49–1.93	0.937								

GSE21653 and GSE42568

	Univariate Analysis				Multivariate Analysis				Multivariate Analysis		
	Hazard Ratio	95% CI	*P* value		Hazard Ratio	95% CI	*P* value		Hazard Ratio	95% CI	*P* value
No. of patients (n=130)				No. of patients (n=130)				No. of patients (n=130)			
No. of events (n=58)				No. of events (n=58)				No. of events (n=58)			
**Immune prognostic index**				**Immune prognostic index**				**Immune prognostic index**			
Continuous				Continuous	1			Low	1		
As score increases	2.47	1.42–4.30	**0.001**	As score increases	2.3	1.31–4.04	**0.004**	High	3.9	1.21–12.51	**0.022**
**Risk groups**											
Low	1										
High	4.32	1.35–13.82	**0.014**								
**Clinical variables**				**Clinical variable**				**Clinical variable**			
Age (years)				LN status				LN status			
≤50	1			Negative	1			Negative	1		
>50	1.36	0.78–2.35	0.279	Positive	2.19	1.25–3.83	**0.006**	Positive	2.25	1.29–3.95	**0.004**
Tumor size (cm)											
≤2	1										
>2	0.89	0.35–2.22	0.797								
LN status											
Negative	1										
Positive	2.41	1.38–4.22	**0.002**								
Histologic grade											
1&2	1										
3	1.54	0.83–2.87	0.171								

Abbreviations: CI, confidence interval, GEO, Gene Expression Omnibus; LN, lymph node; METABRIC, Molecular Taxonomy of Breast Cancer International Consortium
P values < 0.05 are marked in bold

Supplementary Table S3. Comparative analysis of prognostic performance of immune prognostic index and other immune gene signatures in the discovery and validation dataset

Gene signatures or index	Univariate Cox Regression		Kaplan-Meier analysis	Multivariate Cox Regression	
**Discovery dataset (n= 386)**					
	Hazard Ratio (95% CI)	*P* value	Log-rank *P* value	Hazard Ratio (95% CI)	*P* value
Immune Prognostic Index (High vs low)	5.77 (3.40-9.80)	**<0.001**	**<0.001**	4.07 (2.19-7.55)	**<0.001**
Immune Prognostic Index (Intermediate vs low)	2.42 (1.52-3.86)	**<0.001**		2.24 (1.40-3.60)	**<0.001**
B cell response signature (Ascierto et al., *Breast Cancer Res Treat* 2012) [1]	2.37 (1.74-3.22)	**<0.001**	**<0.001**	1.42 (0.85-2.38)	0.176
HRneg/Tneg signature (Yau et al., *Breast Cancer Res* 2010)* [2]	2.02 (1.50-2.72)	**<0.001**	**<0.001**	1.09 (0.69-1.72)	0.712
**Validation dataset (METABRIC, n= 341)**					
Gene signatures or index	Univariate Cox Regression		Kaplan-Meier analysis	Multivariate Cox Regression	
	Hazard Ratio (95% CI)	*P* value	Log-rank *P* value	Hazard Ratio (95% CI)	*P* value
Immune Prognostic Index**	2.16 (1.60-2.91)	**<0.001**	**<0.001**	1.71 (1.16-2.50)	**0.006**
B cell response signature (Ascierto et al., *Breast Cancer Res Treat* 2012)*** [1]	1.83 (1.39-2.40)	**<0.001**	**<0.001**	1.17 (0.80-1.71)	0.431
HRneg/Tneg signature (Yau et al., Breast Cancer Res 2010) * [2]	1.96 (1.5-2.56)	**<0.001**	**<0.001**	1.28 (0.84-1.95)	0.252

*Among 14 genes, 13 genes except *HRBL* and 12 genes except *HRBL* and *ZNF3* were used for analysis in the discovery and METABRIC dataset, respectively.
** Three genes (*TRAT1*, *IL21R*, and *CTLA4*) were used to calculate the immune prognostic index in the METABRIC dataset.
***In case of five-gene signature, four genes except *IGLL5* were used for analysis in the METABRIC dataset.
Abbreviations: CI, confidence interval, METABRIC, Molecular Taxonomy of Breast Cancer International Consortium
P values < 0.05 are marked in bold

Supplementary Table S4. Public datasets used for the discovery and validation datasets

Dataset type	Platform	GEO Number	Year of data availab ility	Sample collection method	No. of patients	No. of events	Surviv al type	References
Datasets used for discovery								
GEO	GPL96	GSE4922	2006	Surgical removal	578	89	DFS	Ivshina et al., *Cancer Res* 2006 [3]
		GSE6532	2006	NA	327	62	DMFS	Loi et al., *J Clin Oncol* 2007 [4]
		GSE7390	2006	Surgical removal	198	62	DMFS	Desmedt *et al., Clin Cancer Res* 2007 [5]
		GSE11121	2008	Surgical removal	200	46	DMFS	Schmidt et al., *Cancer Res* 2008 [6]
		GSE31519	2011	Biopsies before treatment	67	23	DFS	Rody et al., *Breast Cancer Res* 2011 [7]
Datasets used for validation								
GEO	GPL96	GSE17705	2010	Surgical removal	298	70	DMFS	Symmans et al., *J Clin Oncol* 2010 [8]
		GSE3494	2005	Surgical removal	251	55	OS	Lundberg et al., *Clin Cancer Res* 2017 [9]
	GPL570	GSE21653	2010	Surgical removal	266	70	DFS	Patil et al., *Bioinformatics* 2015 [10]
		GSE42568	2013	Biopsies before treatment	104	49, 36	DFS, OS	Clarke et al., *Carcinogenesis* 2013 [11]
METABRIC			2012	Surgical removal	2509	1143	OS	Pereira et al., *Nat Commun.* 2016 [12]

Abbreviations: DFS, disease-free survival; DMFS, distant metastasis-free survival; GEO, Gene Expression Omnibus; METABRIC, Molecular Taxonomy of Breast Cancer International Consortium; OS, overall survival

Supplementary Table S5. List of 37 proliferation-related genes and 110 immune response-related genes

A separate excel file is provided

Supplementary Table S6. Cox regression univariate analysis results for the top 10 significant immune response-related genes in each molecular subtype

HR+/HER2-	coef	hr	se(coef)	z	pvalue
CD69	-0.68986	0.501646	0.211284	-3.26509	0.001094
CD55	-1.02617	0.358375	0.323082	-3.1762	0.001492
TRAF3IP3	-0.93552	0.392382	0.311055	-3.00757	0.002633
EVI2B	-0.91907	0.398891	0.30849	-2.97925	0.00289
IL21R	-0.80724	0.44609	0.273252	-2.95418	0.003135
IGHM	-0.42671	0.652655	0.148793	-2.86779	0.004134
IGJ	-0.38369	0.681341	0.135338	-2.83506	0.004582
CR2	-0.46309	0.629334	0.164106	-2.82192	0.004774
GZMB	-0.553	0.575221	0.20029	-2.761	0.005762
STAP1	-0.59319	0.552562	0.218993	-2.70871	0.006755

HR+/HER2+	coef	hr	se(coef)	z	pvalue
KLRB1	-1.52862	0.216835	0.325476	-4.69656	2.65E-06
PRKCB	-2.19827	0.110995	0.468355	-4.6936	2.68E-06
CD37	-1.93956	0.143767	0.413562	-4.68989	2.73E-06
GPR171	-2.04521	0.129353	0.436535	-4.6851	2.80E-06
CD3D	-1.92875	0.14533	0.417865	-4.61572	3.92E-06
PPP1R16B	-1.67262	0.187754	0.365964	-4.57047	4.87E-06
ITK	-2.57119	0.076444	0.564613	-4.5539	5.27E-06
SH2D1A	-2.41604	0.089274	0.531415	-4.54643	5.46E-06
TNFRSF1B	-3.84986	0.021283	0.847878	-4.54058	5.61E-06
CD48	-2.60977	0.073551	0.582633	-4.47927	7.49E-06

HR-/HER2+	coef	hr	se(coef)	z	pvalue
BANK1	-0.94469	0.3888	0.261254	-3.61599	0.000299
GIMAP6	-2.0162	0.13316	0.644425	-3.12868	0.001756
CD69	-1.61401	0.199087	0.542267	-2.97642	0.002916
GPR18	-0.94085	0.390294	0.335457	-2.80469	0.005036
LY9	-0.99188	0.370877	0.353694	-2.80436	0.005042
VNN2	-1.05607	0.347819	0.388099	-2.72115	0.006506
TCL1A	-2.0106	0.133909	0.742011	-2.70966	0.006735
CYTIP	-1.61108	0.199672	0.606694	-2.6555	0.007919
CTSW	-0.89989	0.406614	0.351074	-2.56325	0.01037
PTPRC	-1.2287	0.292673	0.483899	-2.53916	0.011112

TNBC	coef	hr	se(coef)	z	pvalue
PDCD1LG2	-0.67776	0.507751	0.258882	-2.61804	0.008844
LTA	-1.2035	0.300141	0.484417	-2.48444	0.012976
IGLV1-44	-0.39441	0.674078	0.1651	-2.38891	0.016898
CCR10	-0.75399	0.470485	0.329995	-2.28486	0.022321
TNFRSF9	-0.8434	0.430247	0.370424	-2.27684	0.022796
GPR18	-0.47547	0.621591	0.218	-2.18107	0.029178
IGLJ3	-0.49076	0.612164	0.232956	-2.10664	0.035148
IGHG1	-0.54498	0.579854	0.268435	-2.03021	0.042335
IGHM	-0.29906	0.741512	0.153638	-1.94655	0.051589
CD19	-0.49133	0.611815	0.254689	-1.92912	0.053716

Abbreviations: HER2, human epidermal growth factor receptor 2; HR, hormone receptor; TNBC, triple-negative breast cancer

References

1 Ascierto, M. L. *et al.* A signature of immune function genes associated with recurrence-free survival in breast cancer patients. *Breast Cancer Res Treat* **131**, 871-880, doi:10.1007/s10549-011-1470-x (2012).

2 Yau, C. *et al.* A multigene predictor of metastatic outcome in early stage hormone receptor-negative and triple-negative breast cancer. *Breast Cancer Res* **12**, R85, doi:10.1186/bcr2753 (2010).

3 Ivshina, A. V. *et al.* Genetic reclassification of histologic grade delineates new clinical subtypes of breast cancer. *Cancer Res* **66**, 10292-10301, doi:10.1158/0008-5472.CAN-05-4414 (2006).

4 Loi, S. *et al.* Definition of clinically distinct molecular subtypes in estrogen receptor-positive breast carcinomas through genomic grade. *J Clin Oncol* **25**, 1239-1246, doi:10.1200/JCO.2006.07.1522 (2007).

5 Desmedt, C. *et al.* Strong time dependence of the 76-gene prognostic signature for node-negative breast cancer patients in the TRANSBIG multicenter independent validation series. *Clin Cancer Res* **13**, 3207-3214, doi:10.1158/1078-0432.CCR-06-2765 (2007).

6 Schmidt, M. *et al.* The humoral immune system has a key prognostic impact in node-negative breast cancer. *Cancer Res* **68**, 5405-5413, doi:10.1158/0008-5472.Can-07-5206 (2008).

7 Rody, A. *et al.* A clinically relevant gene signature in triple negative and basal-like breast cancer. *Breast Cancer Research* **13**, R97, doi:10.1186/bcr3035 (2011).

8 Symmans, W. F. *et al.* Genomic index of sensitivity to endocrine therapy for breast cancer. *J Clin Oncol* **28**, 4111-4119, doi:10.1200/JCO.2010.28.4273 (2010).

9 Lundberg, A. *et al.* Gene Expression Signatures and Immunohistochemical Subtypes Add Prognostic Value to Each Other in Breast Cancer Cohorts. *Clin Cancer Res* **23**, 7512-7520, doi:10.1158/1078-0432.CCR-17-1535 (2017).

10 Patil, P., Bachant-Winner, P. O., Haibe-Kains, B. & Leek, J. T. Test set bias affects reproducibility of gene signatures. *Bioinformatics* **31**, 2318-2323, doi:10.1093/bioinformatics/btv157 (2015).

11 Clarke, C. *et al.* Correlating transcriptional networks to breast cancer survival: a large-scale coexpression analysis. *Carcinogenesis* **34**, 2300-2308, doi:10.1093/carcin/bgt208 (2013).

12 Pereira, B. *et al.* The somatic mutation profiles of 2,433 breast cancers refines their genomic and transcriptomic landscapes. *Nat Commun* **7**, 11479, doi:10.1038/ncomms11479 (2016).

Supplementary Figures

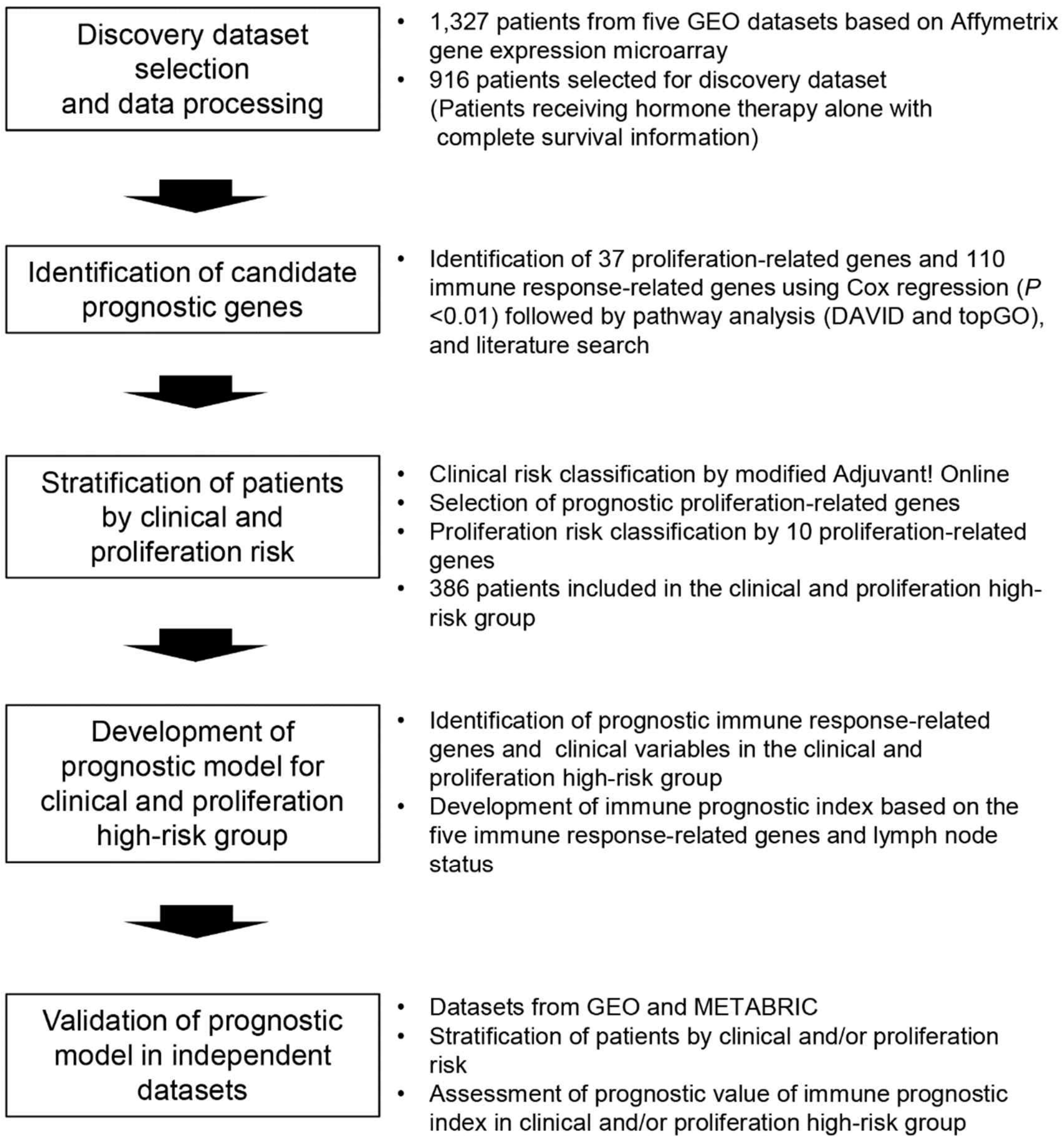

Supplementary Figure S1. Flow chart of this study. GEO, Gene Expression Omnibus; METABRIC, Molecular Taxonomy of Breast Cancer International Consortium.

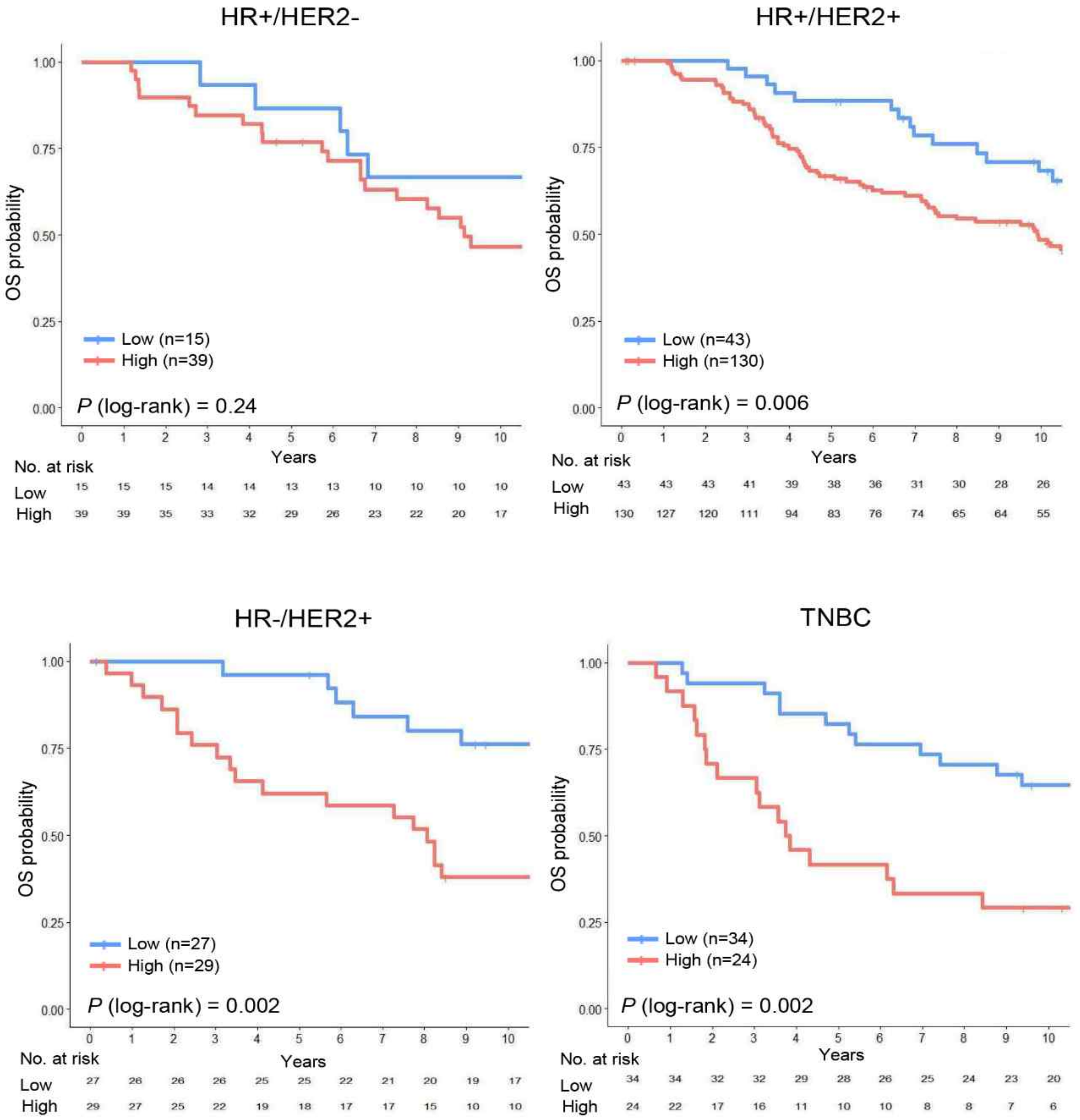

Supplementary Figure S2. Subgroup analysis (according to the immune prognostic index) of the METABRIC validation dataset. Kaplan-Meier plots of overall survival (OS) for patients with HR+/HER2-, HR+/HER2+, HR-/HER2+ breast cancer, or TNBC. Patients were stratified into two risk groups (low risk and high risk) according to the immune prognostic index. Optimal cutoff points for the immune prognostic index used for risk classification were determined using maximally selected rank statistics.

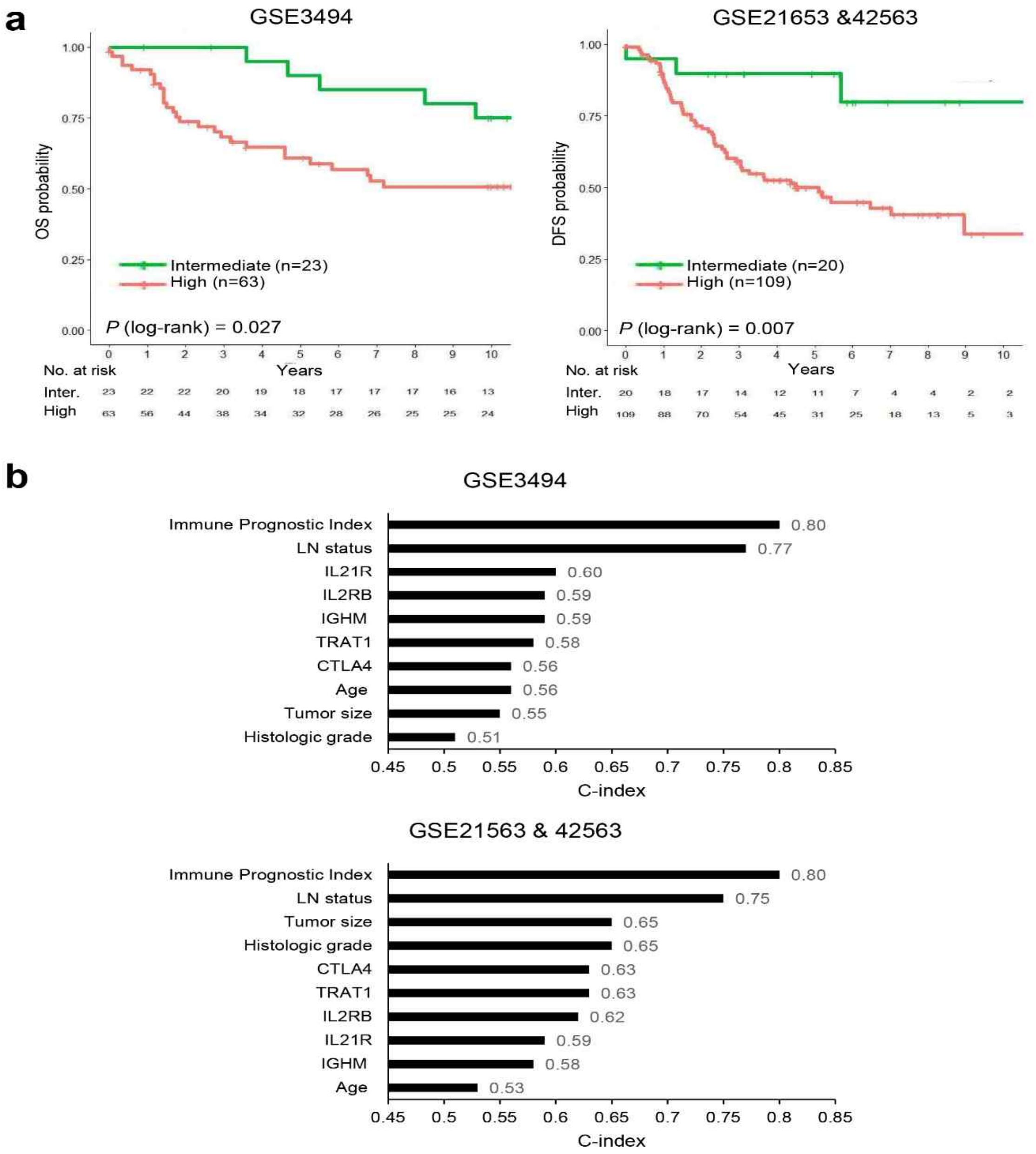

Supplementary Figure S3. Prognostic significance of the immune prognostic index for the validation datasets, including chemotherapy-treated patients. (**a**) Kaplan-Meier plot of overall survival (OS) of patients in the GSE3494 dataset or disease-free survival (DFS) in the GSE21653 and GSE42568 datasets. Patients were stratified into intermediate and high-risk groups according to the immune prognostic index. Optimal cutoff points for the immune prognostic index used for risk classification were determined using maximally selected rank statistics. (**b**) Prognostic performance of the immune prognostic index according to the C-index.

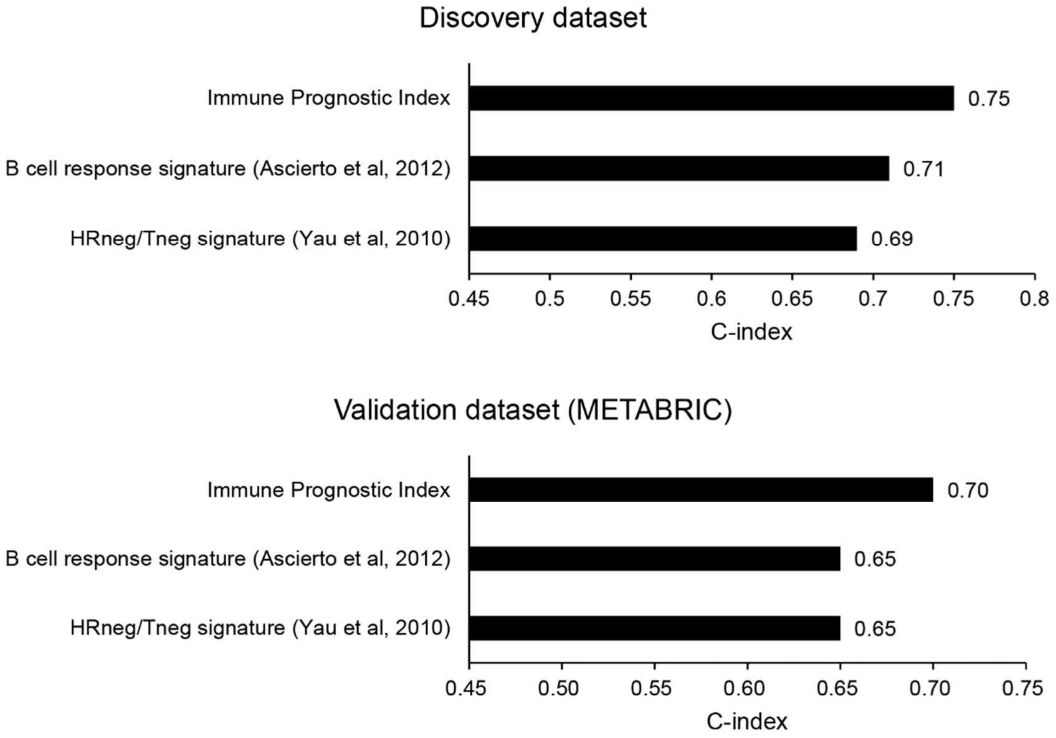

Supplementary Figure S4. Comparison of prognostic performance of the immune prognostic index with other immune gene signatures according to the C-index in the discovery and validation (METABRIC) datasets.

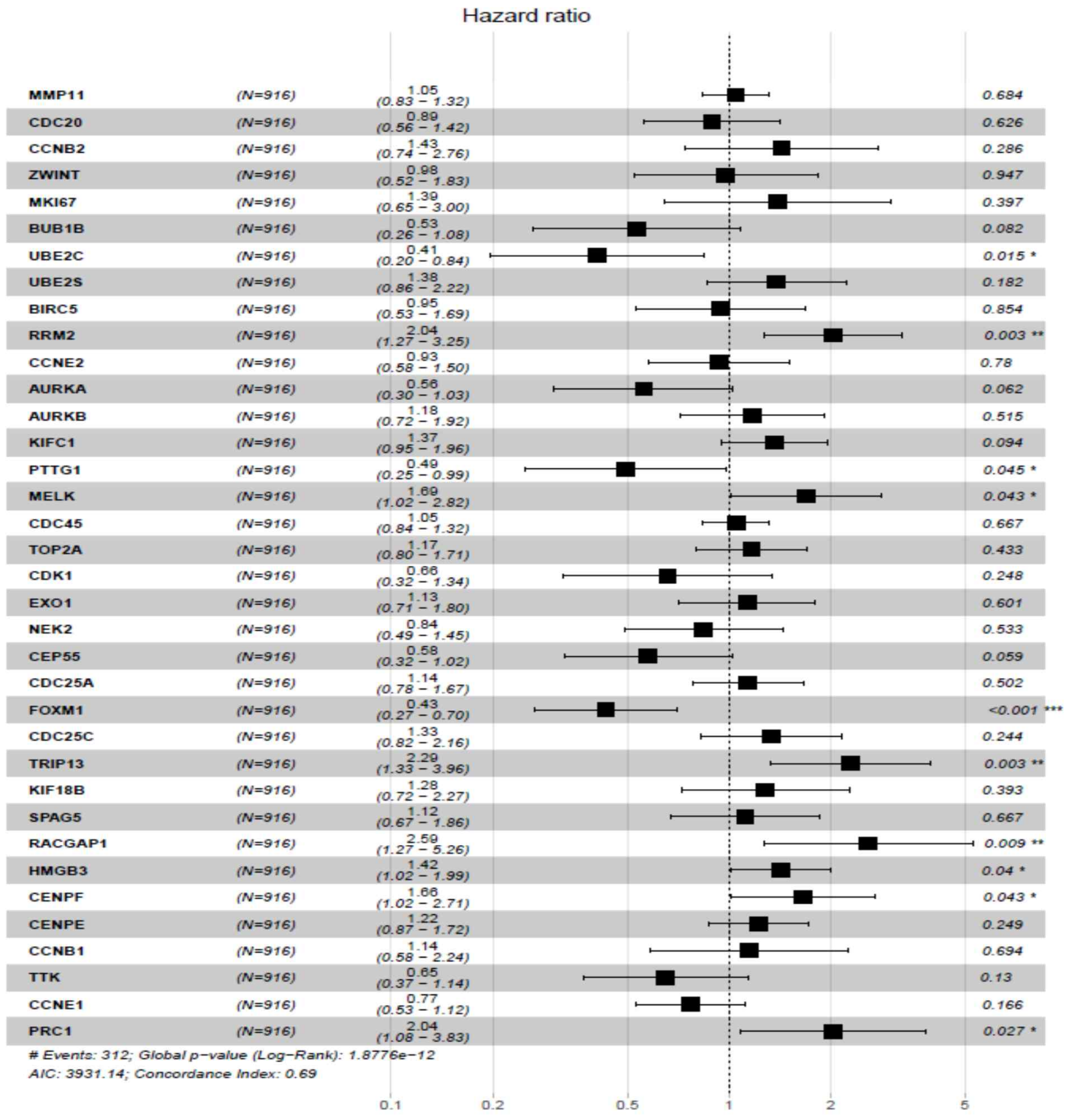

Supplementary Figure S5. Forest plot showing hazard ratios obtained by multivariate cox regression analysis of 37 proliferation-related genes in the discovery dataset. Hazard ratios and 95% confidence intervals are shown on the horizontal axis. **P* value < 0.05.
